# Resource Translocation Modelling Highlights Density-Dependence Effects in Fruit Production at Various Levels of Organisation

**DOI:** 10.3389/fpls.2022.931297

**Published:** 2022-07-08

**Authors:** Michel Génard, Françoise Lescourret, Nadia Bertin, Gilles Vercambre

**Affiliations:** INRAE, PSH, Paris, France

**Keywords:** carbon-based model, density dependence, fruit, mass, scale, source-sink, flow, unloading

## Abstract

The size of fruit cells, seeds and fruits depends on their number. Could this density-dependence effect result from sugar resource sharing and, if so, does it involve phloem sugar flow or the intensity of sugar unloading to the sink? A density-dependence model (DDM) describing these processes was designed and parameterised for six species at five levels of organisation: cells and seeds within fruits, fruits within clusters, fruits within plants and plants within plots. Sugar flow was driven by phloem conductance, determined by parameters *α*, governing the shape of its relationship to population size, and *κ*, its value for a population size of one. Sugar unloading followed Michaelis–Menten kinetics with parameters *V_m_* (maximal unloading rate) and *K_m_* (Michaelis constant). The DDM effectively reproduced the observed individual mass dynamics, the undercompensating density dependence observed in most species at all sub-plant levels and the undercompensating, exact and overcompensating density dependence observed at the plant level. Conductance (*κ*) was a scaling factor varying with the level of organisation. *V_m_* was positively correlated with density dependence, and α was negatively correlated with density dependence only if the plant-within-plot level was not considered. Analysis of the model’s behaviour indicates that density dependence of fruit growth could be a result of sugar sharing, and that both phloem sugar flow and sugar unloading contribute to these effects.

## Introduction

For many wild fruit-bearing species, the production of fruits containing seeds is a key factor in population dynamics. The total fruit mass per tree is frequently the factor best explaining fruit-eating and seed dispersal behaviour by bird and mammals (Jordano, 1995; Floerchinger et al., 2010). Many of the characteristics of fleshy fruits have been interpreted as co-adapted features of plants governing the choice of fruit species by animals. These features include the palatability and nutrient content of edible tissues (Gautier-Hion et al., 1985) and fruit size (Floerchinger et al., 2010) or colour (Valenta and Nevo, 2020). In domesticated species, fruit size is also an important criterion of attractiveness for human consumers, and fruit yield is an essential determinant of the grower’s profit.

Fruit production can be analysed at several levels of organisation. The yield per unit surface area is the product of plant number per unit surface area and yield per plant, which is obtained by multiplying fruit number per plant and fruit mass. Fruit mass is the product of cell number per fruit and cell mass. A population can be defined at each level. For example, a plant or infructescence can be considered as a collection of fruits forming a population, and an individual fruit can be considered as a population of seeds (and associated flesh) or simply as a population of cells.

Population size is highly variable at all levels. In apple, fruit cell number ranges from 30 × 10^6^ to 120 × 10^6^ (Westwood et al., 1967; Goffinet et al., 1995), seed number per kiwifruit ranges from 100 to 1,400 (Lescourret et al., 1998b), St. Lucie’s cherry (*Prunus mahaleb*) bears between 700 and 30,000 fruits per plant (Jordano, 1995) and the density of plants in peach orchards ranges between 400 and 2,000 trees/ha (Hoying et al., 2005).

In general, the various populations respect a simple principle according to which larger populations have a greater total mass and lower masses of individuals (Begon et al., 1996). Fruits also follow this general trend, as shown in many studies on cell number per fruit (Tuberosa et al., 1992; Cowan et al., 1997; Higashi et al., 1999; Jullien et al., 2001; Bertin et al., 2009) and fruit number per plant (Johnson and Handley, 1989; Corelli-Grappadelli and Morandi, 2012). The concept of density dependence (Hixon and Johnson, 2009) can be used to describe this phenomenon.

Simple models describing the effect of population size on individual and population masses have been proposed by Lescourret and Génard (2003), Quilot and Génard (2008) and Prudent et al. (2014). By applying these models to various case studies, the authors found that undercompensating density dependence is encountered in most cases. Undercompensation applied to the case of mass means that the decrease in individual mass with increasing population size is insufficient to limit the increase in population mass. From a theoretical point of view, undercompensation is one of three possibilities. The other two possibilities are exact compensation, in which the mass of the population remains constant, and overcompensation, in which a competitive effect results in a decrease in the mass of the population with increasing size (Begon et al., 1996). Lescourret and Génard (2003) showed that the intensity of density dependence varies considerably with the level of organisation. They observed strong density dependence for populations of seeds and their associated flesh in kiwifruit and apple, and weak density dependence for populations of fruits grouped into clusters, such as grapes, red dogwood or common ivy berries. Prudent et al. (2014) showed that the model parameter reflecting the level of density dependence varied with the availability of assimilates. Explorations of the role of assimilates must go beyond the initial, phenomenological model, and make use of process-based models.

We designed a process-based model for testing the hypothesis that density dependence of individual and population fruit masses results from carbon resource sharing during growth. The density-dependence model (DDM) represents the transport of assimilates from a source to a sink, as proposed by Thornley and Johnson (1990), Minchin et al. (1993) and Bruchou and Génard (1999). It first describes the source-to-sink flow of assimilates in the phloem, and then considers their active unloading at the sink. The sinks investigated in this study corresponded to different levels of organisation: cell, seed plus associated flesh, individual fruit and total fruit per plant. We used experimental data for tomato cells to determine how the DDM depicts the temporal dynamics of population mass. We then investigated the ability of the DDM to simulate density-dependent effects on growth at various levels of organisation and for different fruit-producing species. Finally, we used this model to study the relative effects on density dependence of assimilate flow in the phloem and unloading in fruit tissues.

## Materials and Methods

### Plant Material and Measurements

The data were collected at five levels of organisation: the cell, the seed (with the associated flesh), the fruit within a cluster, the fruit within a plant and the plant (i.e., the set of all the fruits of the plant). For calibration of the model at these five levels, we used both original observations and published data. In total, we used 12 datasets relating to six fruit-bearing species.

Cell numbers were evaluated in 2000–2007, at INRAE Avignon, on the mesocarp of 22 mature peach fruits [*Prunus persica* (L.) Batsch^1^; Alexandra Cv.] grown at two fruit loads (1 and 6 fruits per fruit-bearing shoot) and on the pericarp of 137 tomato fruits (*Solanum lycopersicum* L.; Raissa, Levovil and Cervil Cv.) throughout the season. Bertin (2005) showed that the number of cells in the tomato pericarp varied with the position of the fruit in the truss (cluster of fruits). We therefore studied three positions (first, third and fifth fruits from the base of the truss) in the Raissa cultivar. For the Cervil (cherry tomato) and Levovil (large tomato) cultivars, we varied the number of cells per truss by manipulating the number of fruits: 5 and 20 fruits per truss for Cervil and two and six fruits per truss for Levovil. The number of cells was measured by dissociating the cells in the fruit tissue, as described by Bertin et al. (2002) and based on the method of Bünger-Kibler and Bangerth (1983). The mean cell mass per fruit was estimated by dividing the mass of the mesocarp for peach or the pericarp for tomato by the number of cells.

The number of seeds per fruit and fruit mass were measured in 1999–2002 on 300 mature apple fruits (*Malus domestica* Borkh.; Royal Gala and Granny Smith Cv.) grown at INRAE Avignon and 50 sarsaparilla (*Smilax aspera* L.) fruits harvested at maturity in the Mediterranean forest close to INRAE Avignon.

Fruit number and fruit mass per berry were measured in 2002 on 30 grape (*Vitis vinifera* L.; Muscat Cv.) and 21 common dogwood (*Cornus sanguinea* L.) clusters harvested at maturity from the vineyard (for grape) and the nearby Mediterranean forest (for dogwood). The number of fruits per tree and mean individual fruit mass were assessed for peach (Suncrest Cv.) in 1985–1987 on 185 trees (Suncrest Cv.) grown at INRAE Avignon and taken for apple (Summerred Cv.) from Hansen’s (1989) observations.

The number of trees and yield per tree were taken from the work of Hutton et al. (1987) on 45 peach (Yanco queen Cv.) orchards.

### Density-Dependence Model

**Figure 1:**
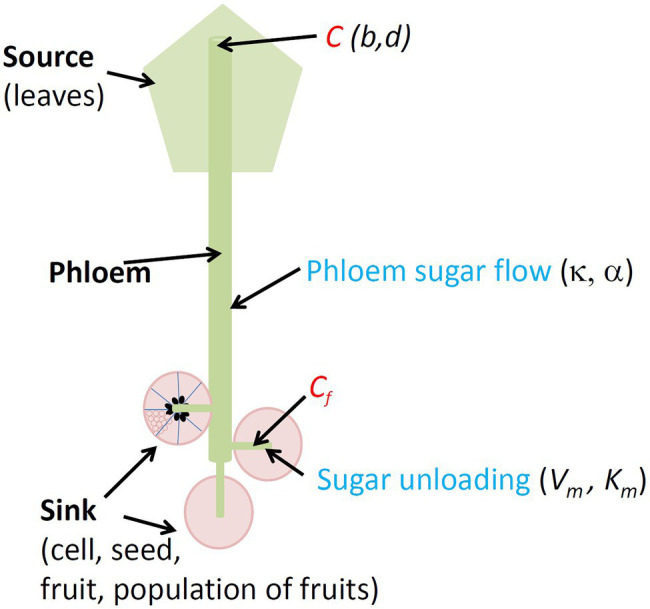
Conceptual diagram of the density-dependence model (DDM). Objects (in bold black), sugar concentrations (in red) in plant phloem (*C*) and in sink phloem (*C_f_*), sugar transport (in blue) and corresponding model parameters (in italics) are indicated.

The components of the system studied were the source of sugars (plant leaves), the phloem transfer pathway between the source and the sink and the sink for sugars (Figure 1). The sink is defined as a population of *n* individuals, which may be cells, seeds plus associated flesh, fruits or the total fruit production of the whole plant. We used the model described by Thornley and Johnson (1990), in which the translocation of sugars in the phloem is represented according to Münch’s theory of solution flow. Münch’s theory has two main components: the creation of turgor pressure in the phloem by an osmotic gradient that draws water from the xylem to the phloem because of a high concentration of sugars in the source and a movement of sugars by mass flow in the phloem from source to sink. It remains the best-supported theory to explain phloem transport although it has been challenged (De Schepper et al., 2013; Knoblauch and Peters, 2017). In particular, the results of Knoblauch et al. (2016) on long-distance transports have provided strong support to this theory.

As done by Minchin et al. (1993), we assumed that phloem turgor pressures are approximately proportional to sugar concentrations (Minchin et al., 1993). On this basis, the flow of sugars (*F*, g h^−1^) from the source to the sink may be described as follows:


(1) $$F=kC\left(C-C_{f}\right)$$

where *C* and *C_f_* (g cm^−3^) are the phloem sugar concentration of the source and of the sink, respectively, and *k* (cm^6^ g^−1^ h^−1^) is the conductance of the transfer pathway to the *n* individuals of the sink.

With more fruits per plant, there are also more vessels feeding this collection of fruits. We assume that this logic applies at each organisational level. We therefore assumed that conductance *k* increases with the number of sinks *n*, as follows:


$$k=\kappa \;n^{\alpha }$$

with *κ* (cm^6^ g^−1^ h^−1^) the conductance value for a population of one single individual and α (dimensionless) a parameter accounting for the shape of the positive relationship between *n* and *k*, the simplest cases being those in which conductance is proportional to population size with *α* = 1, or independent of population size with *α* = 0.

The unloading of the sugars at the sink (*U*, g h^−1^) was assumed to follow Michaelis–Menten kinetics, as proposed by Minchin et al. (1993) and Fishman and Génard (1998), adapted for a population of size *n*:


(2) $$U=\frac{\mathrm{ns}V_{m}C_{f}}{\left(K_{m}+C_{f}\right)}$$

where *s* (g) is the dry mass of an individual of the population, *V_m_* (g sugars g dry mass^−1^ h^−1^) is the maximal unloading rate per unit dry mass and *K_m_* (g cm^−3^) is the Michaelis–Menten constant.

The assimilate concentration in the phloem of the sink (*C_f_*) was calculated assuming an absence of storage capacity within the phloem, such that the total assimilate flow through the transfer pathway matches that unloaded at the sink. Solving *F = U* gives:


(3) $$C_{f}=\frac{\kappa n^{\alpha }\left(C-K_{m}\right)C-\mathrm{ns}V_{m}+\sqrt{\begin{array}{l} {\left(\kappa n^{\alpha }\left(K_{m}-C\right)C+\mathrm{ns}V_{m}\right)}^{2}\\ +4\kappa^{2}n^{2\alpha }K_{m}C^{3} \end{array}}}{2\kappa n^{\alpha }C}$$

Interestingly, if *α* is equal to 1, *C_f_* is independent of *n*, and, according to Equation 2, the flow of sugars per individual $\left(\frac{U}{n}\right)$ is constant, which implies that there is no density dependence. An absence of density dependence is also found when *K_m_* < < *C_f_*, a situation in which $\frac{U}{n}\sim sV_{m}$ .

Combining Equations 2 and 3 and integrating Equation 2 over time yields the dry mass of an individual:


$$s\left(t\right)=s_{0}+\frac{\left(1-r\right)}{n}\overset{t}{\underset{t_{0}}{\int }}\;Udt$$

where *t*_0_ is the time (h) after which the size of the population remains constant: the end of cell division for the cellular level, of anthesis for the seed level and of fruit thinning for the fruit and plant levels in the case of fruit crops. *s*_0_ is the individual dry mass at *t*_0_, and *r* is the proportion of sugars supplied to the sink used for its respiration.

The fresh mass of an individual (*M*, g) and the total fresh mass of the population (*TM*, g) were calculated as follows:


$$M\left(t\right)=\frac{s\left(t\right)}{DMC}\;\;TM\left(t\right)=n\;M\left(t\right)$$

where *DMC* is the dry matter content of sink tissues.

**Figure 2:**
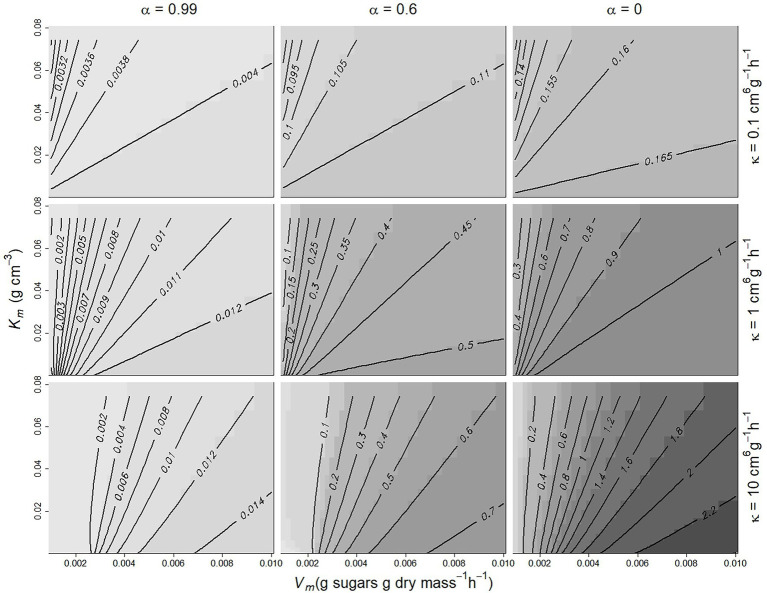
Contour plot of the variation of the density-dependence index $\left(DD=-\;\frac{M_{n_{\text{min}}}-M_{n_{\text{max}}}}{\bar{M}}\times \frac{\bar{n}}{n_{\text{min}}-n_{\text{max}}}\right)$ as a function of *α*, *κ*, *V_m_*, and *K_m_*. DD increases from light grey to black. Population size ranged from 1 to 20, and growth duration was 50 days, with *C* = 0.1 g cm^−3^, *s*_0_ = 3 g, *DMC* = 0.16.

Density dependence (DD) applied to the case of fruit mass can be represented by a positive index quantifying the increase in sensitivity of *M* at fruit maturity as population size *n* increases from *n_min_* to *n_max_* $\left(DD=-\;\frac{M_{n_{\text{min}}}-M_{n_{\text{max}}}}{\bar{M}}\times \frac{\bar{n}}{n_{\text{min}}-n_{\text{max}}}\right)$ . Figure 2 shows this index as a function of *K_m_* and *V_m_* for three different values of *κ* and *α*. DD is logically close to zero when *α*~1 (*C_f_* is independent of *n*, see Equation 3) and increases as *α* decreases. It also increases with *κ* and *V_m_* and decreases with increasing *K_m_*, which controls the assimilate demand per individual.

### Inputs and Modelling Technique

The main input of the model, population size, was highly variable, ranging from less than 10 at seed level to several million at cell level. The input variable individual initial dry mass *s*_0_ varied with starting date *t*_0_, species/cultivar and level.

The dry matter content of fruit tissue was taken from our own databases or from published studies. It ranged from 0.06 to 0.24 for tomato cv. Levovil and apple cv. Granny Smith, respectively. The value of parameter *r* was estimated at 0.16 from the data for fruit respiration provided by Dejong and Walton (1989), Walton and Dejong (1990), Marcelis and Hofman-Eijer (1995) and Walton et al. (1999).

For the sake of simplicity, at sub-plant levels, the assimilate concentration of the leaf phloem (*C*) was set at a constant value of 0.1 g cm^−3^, which is within the range of 0.05–0.15 g cm^−3^ observed for many species (Dale and Sutcliffe, 1986). At the plant level, we assumed that *C* decreased with plant density due to competition for light. The following equation represents the various shapes of the decreasing relationship:


$$C=\frac{0.1}{1+e^{b\left(n-d\right)}}$$

where *b* and *d* are parameters, *b* being proportional to the rate of decrease in concentration at *d*, which is the density of plants at maximal rate of decrease.

A time step of 1 h was used in the numerical integration. The computer program was written in R simulation language (Venables et al., 2022). The differential equations were solved numerically by the first-order Runge–Kutta method.

Parameter values that could not be obtained from previous publications (*V_m_*, *K_m_*, *κ* and *α* for every level, and *b* and *d* at plant level) were estimated for each of the 12 datasets by fitting the model response (individual masses) to either mass observations throughout the season or mass observations at fruit maturity for different population sizes. The fitting procedure used the genetic algorithm rbga from the R package ‘genalg’ (Willighagen and Ballings, 2015). This algorithm optimises problems by iteratively trying to improve a candidate solution with respect to a performance index (in this study, the sum of squared errors). For each dataset, estimation was performed at least 15 times, yielding a total of 243 values of the parameter vector. For each dataset, the estimate with the best performance index was used for graphical comparisons of the modelled and observed data and to evaluate the goodness-of-fit according to a widely used criterion: relative root mean squared error (RRMSE), the mean difference between simulated and observed results (Kobayashi and Salam, 2000). Smaller values of RRMSE indicate a better fit.


$$\mathrm{RRMSE}=\frac{1}{\bar{X}}\sqrt{\frac{\sum_{i=1}^{N}{\left(Y_{i}-X_{i}\right)}^{2}}{nb}}$$

where *Y_i_* is the predicted value of individual mass *i*, *X_i_* is the measured value, *nb* is the number of data and $\bar{X}$ is the mean of all observed values.

We used the 243 values obtained for each parameter to analyse the variation of the parameter according to the organisation level and species/cultivar, according to classical graphical and statistical techniques: boxplots, analysis of variance and PCA.

## Results

### Parameter Estimation

**Figure 3:**
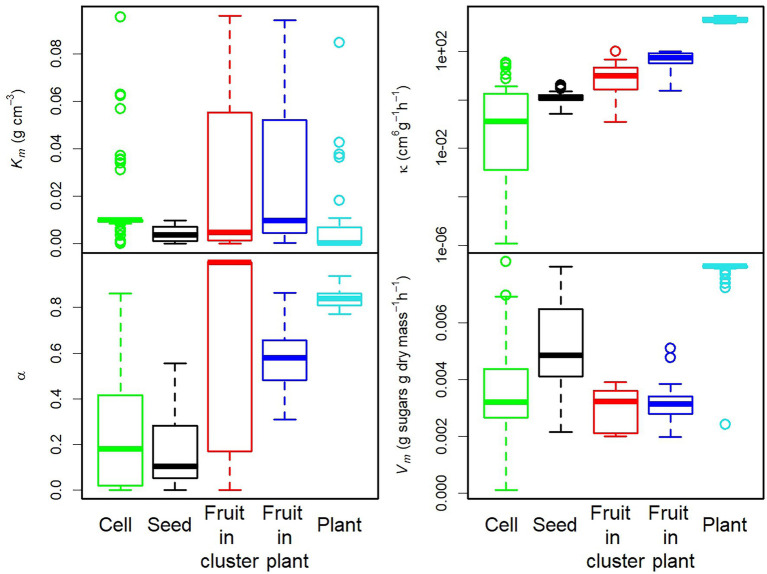
Box plot of the parameter values estimated for each level of organisation. A logarithmic scale was used for parameter *κ*.

The distribution of each parameter value, shown in Figure 3 and Supplementary Figure 1, varied considerably with the level of organisation and species. The parameters *κ*, *α*, and *V_m_* seemed to be more sensitive to organisation level, whereas *K_m_* seemed to be more sensitive to species (Supplementary Table 1).

**Figure 4:**
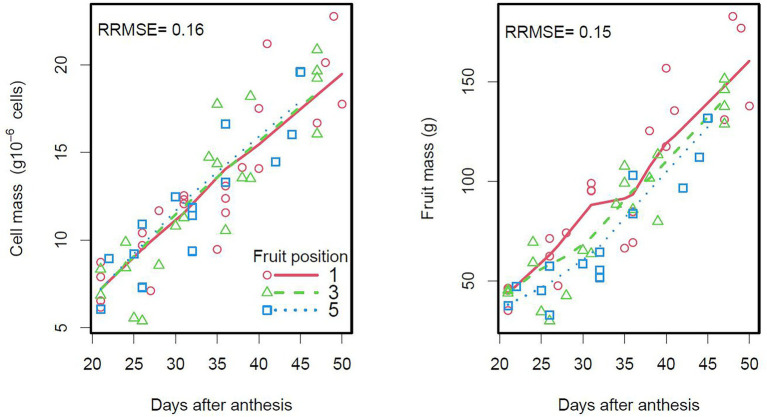
Seasonal variation of cell and fruit masses according to tomato (Raissa Cv.) fruit position in the truss (first, third or fifth fruit from the base of the truss). The points are measured data and the lines are smoothings of DDM outputs. On the right plot, each fruit has a given number of cells (model input based on observations).

**Figure 5:**
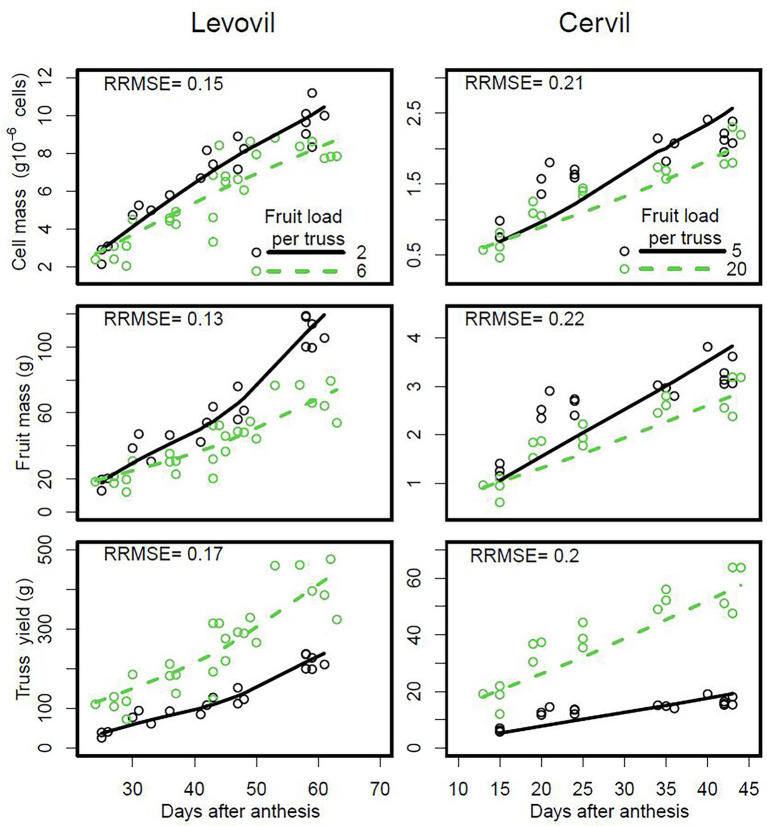
Seasonal variation of cell and fruit masses and total yield of trusses, according to the tomato fruit load in the truss for the cultivars Levovil and Cervil. The points are measured data and the lines are smoothings of DDM predictions. On the plots of the second and third line, each fruit or truss, respectively, has a given number of cells or fruit, respectively (model inputs based on observations).

**Figure 6:**
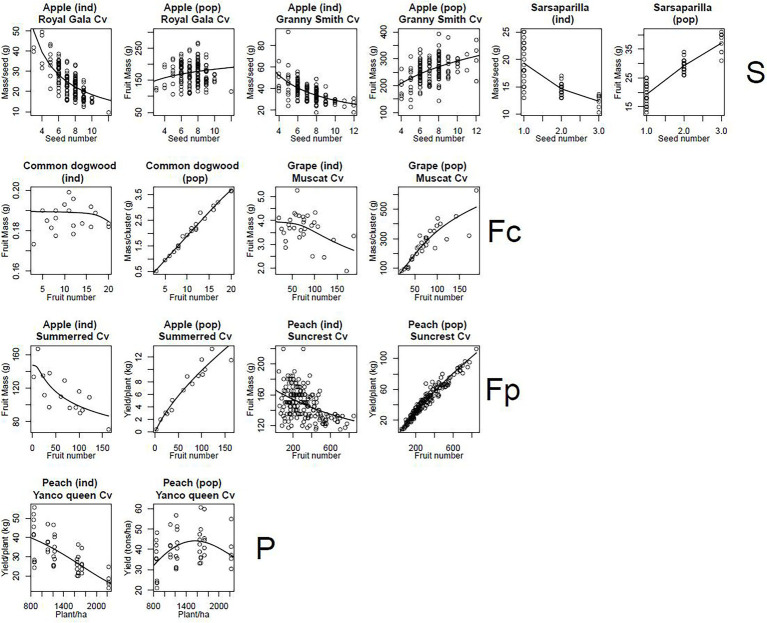
Plots of fruit mass at fruit maturity per individual (ind) and total fruit mass of the population (pop) against number of individuals in the population. The circles correspond to experimental data and the lines are model predictions based on the parameter estimates shown in Supplementary Table 1. The levels of organisation considered were the seed (S), the fruit in a cluster (Fc) or in a plant (Fp) and the plant (P). For each graph, the species studied is indicated, together with variety, if applicable.

**Figure 7:**
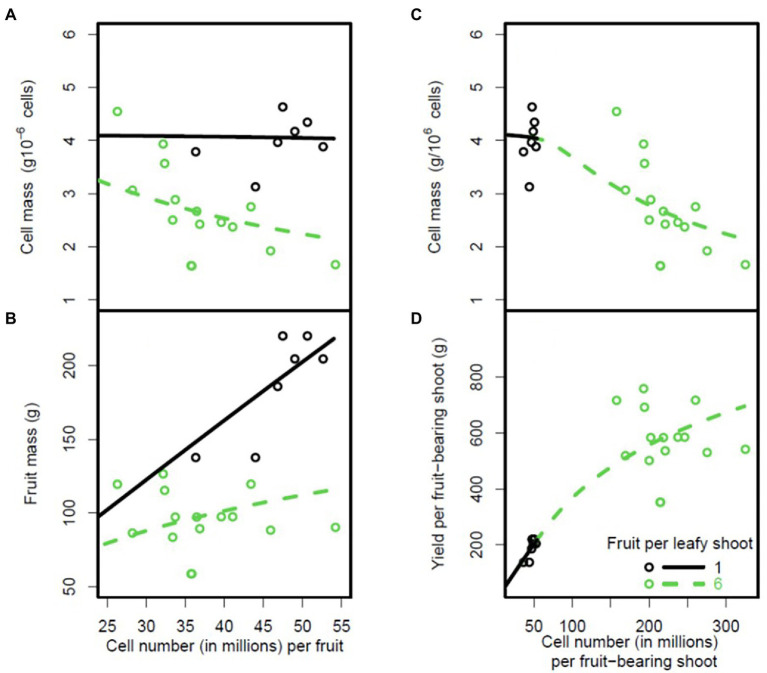
Plots of cell mass and mass of peach fruit mesocarp at fruit maturity against number of cells in the fruit mesocarp **(A,B)** for two fruit loads. Plots of cell mass and total mass per fruit-bearing shoot against number of fruit cells on the shoot **(C,D)**. The circles are the experimental data and the lines are model predictions based on the parameter estimates shown in Supplementary Table 3. The black circles and thick lines correspond to a fruit load of one fruit per leafy shoot and the open circles and thin lines correspond to a fruit load of six fruits per leafy shoot.

Phloem conductance parameters varied over a very wide range, from 3 × 10^−4^ to 1 for *α* and from 1.2 × 10^−6^ to 3 × 10^+3^ cm^6^ g^−1^ h^−1^ for *κ*. As expected, *κ* was strongly related to individual mass at fruit maturity (*M*), serving as a proxy for the number of vessels feeding the individual, according to the relationship *κ* = *M*^0.74^ (Supplementary Table 2). The maximal unloading rate per unit dry mass, *V_m_*, ranged between 0.0001 and 0.00813 h^−1^, consistent with published findings (Patrick, 1994; Ruan and Patrick, 1995; Ruan et al., 1997). The Michaelis–Menten constant *K_m_* varied over a larger range, from 1 × 10^−5^ g cm^−3^, as reported by Rausch (1991) for sugar carriers for which the unloading rate can reach *V_m_* even at low concentrations, to 0.09 g cm^−3^, a value similar to the *K_m_* measured for tomato tonoplasts (Milner et al., 1995). The parameter values selected for the construction of Figures 4–7 correspond to the smallest RRMSE values obtained (Supplementary Table 3).

### The DDM Predicts the Temporal Dynamics of Fruit Masses at Different Levels of Organisation

The ability of the DDM to simulate tomato growth was assessed at different levels of organisation, with the sugar concentration of the plant phloem (*C*) kept constant, at 0.1 g cm^−3^, whatever the day, level and cultivar.

The DDM fitted the seasonal variation of observed cell and fruit masses well, in terms of cell populations in the Raissa cultivar (Figure 4). Consistent with the results of an analysis of variance on the experimental data (Supplementary Table 4), the DDM showed that cell growth did not differ according to fruit position and that fruit mass was higher for proximal than for distal fruits in the truss, due to a larger number of cells per fruit (7.2 × 10^6^ vs. 5.7 × 10^6^ cells).

The DDM adjusted well to the seasonal variation of cell and fruit masses measured in the experiment performed with tomato cultivars Cervil and Levovil, in which the size of the population of cells per truss was manipulated by varying fruit load. It was possible to simulate the effect of fruit load and its interaction with fruit age correctly (Supplementary Tables 5 and 6). Consistent with the experimental data, the DDM predicted a decrease in the mass of cells and fruits with increasing fruit load (Figure 5). By contrast, but also consistent with experimental data, the DDM predicted an increase in total fruit mass on the truss with increasing fruit load per truss, for both cultivars, due to the increase in the total number of cells per truss (from 17 × 10^6^ to 43 × 10^6^ for Levovil and from 8 × 10^6^ to 30 × 10^6^ for Cervil).

### The DDM Explains the Observed Density-Dependence Patterns

For levels above that of the cell, individual mass systematically decreased with increasing population size, except at fruit level for common dogwood (Figure 6). Conversely, except at plant level for peach, population mass systematically increased with population size (Figure 6). In peach, a decrease in sugar concentration (*C*) in the phloem of the plant with increasing plant density (Supplementary Figure 2) explained the bell-shaped curve observed for population mass. All these trends were well simulated by the DDM with RRMSE values between 0.05 and 0.23.

We also analysed density dependence at cell level, using data from the experiment performed on peach trees with two fruit loads (1 and 6 fruits per fruit-bearing shoot; Figure 7). The DDM correctly predicted the increase in fruit mass with the number of cells per fruit (RRMSE = 0.18; Figure 7B). The DDM clearly predicted a decrease in cell mass at fruit maturity with increasing number of cells per fruit for high fruit loads, and a constant cell mass for low fruit loads (Figure 7A, RRMSE = 0.2). Thus, cell mass became density-independent when the carbon supply per fruit increased because of a low fruit load. In this case, the concentrations of sugar in the phloem of the fruit (*C_f_*) were always much higher than the affinity constant (*K_m_*), as illustrated in Supplementary Figure 3. A similar result was obtained for increases in the carbon concentration of the plant phloem (Supplementary Figure 4).

The model correctly predicted the global density-dependence effect observed when cell mass and total fruit mass per fruit-bearing shoot were plotted against the number of cells per fruit-bearing shoot, with a decrease of cell mass with increasing total number of cells per fruit-bearing shoot (RRMSE = 0.2; Figure 7C) and an increase in total fruit mass per shoot (RRMSE = 0.25; Figure 7D).

**Figure 8:**
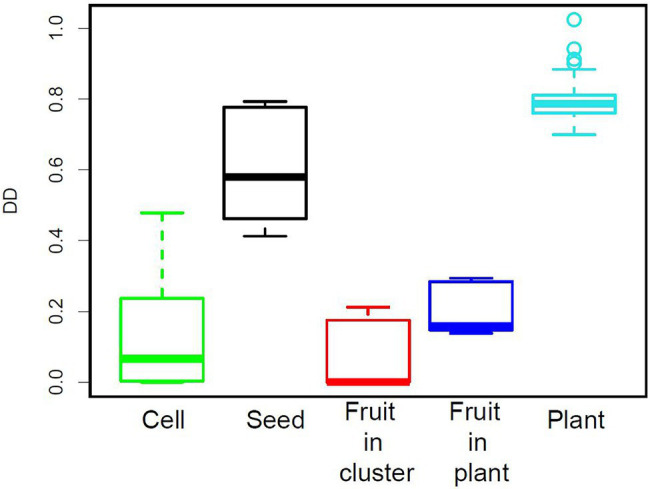
Density-dependence index (DD) for the five levels of organisation considered.

The density-dependence index (DD) was clearly higher at the seed and plant levels than at the cell and fruit levels for the 12 datasets studied here (Figure 8). A similar result was obtained with other datasets (Supplementary Figure 5).

### Phloem Conductance and Sugar Unloading Parameters Vary With the Level of Organisation and Density Dependence

**Figure 9:**
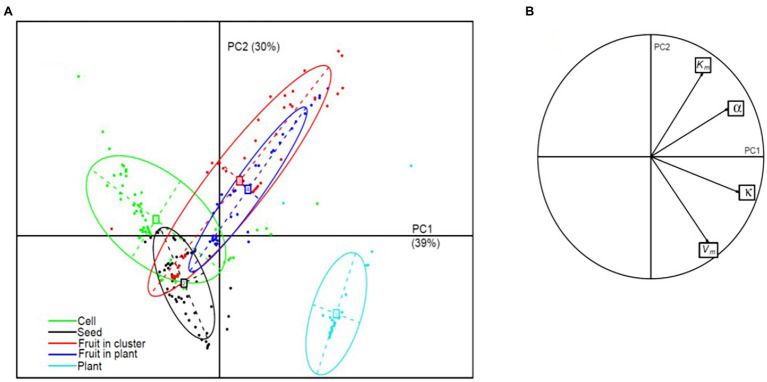
PCA on phloem flow and sugar unloading parameters. **(A)** Projection, in the plane of the first two principal components, of the 243 sets of parameters grouped by organisation level, represented as inertia ellipses (encompassing about 75% of the points); the percentage of the variance explained by each component is indicated in brackets. **(B)** Parameter co-ordinates normed to the square root of the eigenvalue on the correlation circle.

PCA on the 243 sets of parameters estimated in this study showed that both phloem flow and sugar unloading parameters were strongly correlated with the first two components (Figure 9). Cells, seeds, fruits and plants were clearly differentiated on the PC1xPC2 plane, whereas fruits in clusters or on plants were not (Figure 9). The first component (39% of the variance) clearly ordered the organisation levels from the cell level, characterised by low κ and α values (Figure 3), to the fruit and plant levels. The second component (30% of the variance) discriminated the levels of seed and plant, characterised by high *V_m_* and low *K_m_* values (Figure 3), corresponding to a high sink strength, from the cell and fruit levels. The seed and plant levels displayed strong density dependence (Figure 8). These findings therefore suggest that density dependence may be controlled by sink strength. This hypothesis is supported by the finding (Supplementary Figure 6) that, for our datasets, the density-dependence index was correlated with *V_m_* ( r=+0.73 ) and *K_m_* ( r=−0.34 ). However, if plant level was not included in the analysis, we also observed a negative correlation between the density-dependence index and *α* ( r=−0.57 ). At plant level, sugar concentration *C* in the plant decreased with population size, and the estimates of *α* (close to 1) corresponded to non-limiting values of phloem conductance.

In common dogwood fruits, for which we identified no density-dependence effect, 97% of the solutions provided by the genetic algorithm for parameter estimation gave an *α* close to one (positive co-ordinates on PC1 and PC2), potentially accounting for the lack of density dependence. However, 3% of the solutions gave an *α* value close to zero with no density-dependence effect. In this case, low *V_m_* values were obtained, allowing the phloem concentration in the fruit *C_f_* to be much higher than *K_m_* values, rendering *K_m_* negligible in the face of *C_f_* (Supplementary Figure 7). In this situation, the rate of sugar unloading per individual was density-independent because it was about equal to *V_m_* multiplied by the individual mass (Equation 2).

## Discussion

### The DDM Sheds Light on Density Dependence Across Fruit Organisation Levels

Using different fruit-bearing species/cultivars and levels of organisation, we demonstrated that source-sink theory is a powerful framework for explaining the observed patterns of density dependence in the case of mass. The DDM, describing the flow of sugars from the source to the sink and their unloading at the sink, demonstrated a predomination of undercompensation, but at different intensities at different levels of organisation.

Detailed studies of leaf cell populations have clearly demonstrated the existence of a density-dependence phenomenon. They have shown that a decrease in the number of cells can induce an increase in cell volume, sometimes resulting in the maintenance of a constant leaf size. This phenomenon is known as ‘compensation’ (Tsukaya, 2008). Recent studies have shown that this compensation can be explained within the framework of neocellular theory (Beemster et al., 2003; Tsukaya, 2003), through control at organ level and the need for communication between cells (Hisanaga et al., 2015). In a previous study, we showed that cell size in tomato fruits is at least partially controlled at the organ level, through the switch between symplastic and apoplastic sugar transport (Baldazzi et al., 2019). For the sake of simplicity, we did not separate these two types of transport in this study. We also observed an organ-wide control of phloem conductance, which varied with the total number of cells present in the fruit. However, by contrast to the situation in leaves, this control was unable to mediate exact compensation. The presence of a larger number of cells may result in the cells present in the fruit being smaller, but the fruit is always bigger.

According to the DDM, density dependence of fruit units masses results from mechanisms involving the sharing of sugars between individuals in the population. The cells share a sugar supply from the plant phloem, and, thus, the smaller the population, the greater the supply available to each cell. Leaves photosynthesise, producing their own supply of carbohydrates, and the number of chloroplasts per cell and cell size co-vary (Kawade et al., 2013), such that carbohydrate supply per cell increases with decreasing cell number. As a result, leaf area can be kept constant as the number of cells decreases. Conversely, we have shown that, in sink organs, such as fruits, carbohydrate intake per cell increases only slightly with decreasing cell number, resulting in little variability of cell size (especially for a low fruit load), with fruit size strongly positively correlated with cell number (Bertin, 2005; Hammami et al., 2011; Rosati et al., 2011). This may reflect an effect of plant breeding, because Quilot and Génard (2008) showed that the percentage of wild peach genes in the peach genome appeared to increase density dependence.

We found a strong trade-off between seed number and seed mass (seed plus associated flesh). Similar findings have already been reported for interspecific (Jakobsson and Eriksson, 2000) and intraspecific studies (Prudent et al., 2014). This trade-off is important for ecologists interested in survivorship during plant dispersal (Westoby et al., 1996) and establishment (Moles and Westoby, 2004). It has been the subject of theoretical studies since the 1970s focusing on plant fitness and considering the energy or photosynthate invested in seed production (Smith and Fretwell, 1974; Mironchenko and Kozlowski, 2014). Using the DDM, we found that the strong density-dependence effect observed at the seed level was related to the high potential sink strength of seeds (high *V_m_*). Thus, the sources may provide too little sugar if the number of seeds increases. This result is consistent with those of Prudent et al. (2014) and Lázaro and Larrinaga (2018), who showed that the trade-off between seed size and number operates in situations of resource limitation.

Density dependence was particularly weak at the fruit-within-a-cluster level, consistent with the results of Antognozzi et al. (1991) for kiwifruit and those of Lázaro and Larrinaga (2018) for Scandinavian plants. At this level, we found an *α* close to one in many cases, indicating that the total conductance of the cluster was proportional to its number of fruits: this explains the absence of competition. However, the model showed that, in other cases, plants could adopt a completely different strategy, with an *α* value well below one, in which the weak density dependence could be explained by the low *V_m_* and *K_m_*, allowing the fruit to grow continuously to its potential, defined by *V_m_* and therefore independent of density.

At whole-plant level, fruit growers are well aware that individual fruit mass decreases and that yield per plant increases with increasing fruit load (Elfving and Schechter, 1993; Naor et al., 1997). The DDM simulated this phenomenon correctly. Our hypothesis of carbon sharing between fruits is widely accepted in the scientific community and is the foundation of many complex models based on a representation of source-sink relationships for carbon (Buwalda, 1991; Grossman and Dejong, 1994; Lescourret et al., 1998a), some of which take transport within the plant into account (Bruchou and Génard, 1999; Miras-Avalos et al., 2011).

We found undercompensation at all levels other than the plant level, for which we found an exact compensation for mean plant densities (1,200–1,800 trees/ha) and overcompensation for higher densities. Exact compensation has often been reported by plant ecologists, leading to the so-called ‘law of constant final yield’ (Kira et al., 1953). However, these studies were restricted to analyses of differences in total biomass yield with plant density. Giulivo et al. (1984) found overcompensation in peach production in high-density plantations (>1,250 trees/ha), probably because of the mutual shading of leaves and greater respiratory costs. Génard et al. (2000) showed that peach trees planted at a density of 2,500 trees/ha had photosynthesis rates only 50%–70% of those of isolated trees. They also showed that photosynthesis rates/ha increased with tree density, but much more slowly for densities above 1,000 trees/ha. The decrease in photosynthesis rates and the increase in respiratory cost with increasing tree density were modelled in the DDM by considering the decrease in plant phloem concentration (*C*), which could account for overcompensation at high tree densities.

### Phloem Conductance *κ* Is a Scaling Factor, but Density Dependence Seems to Be Controlled Principally by the *α* and Sugar Unloading Parameters

Parameter *κ*, which represents the phloem conductance corresponding to a population consisting of one individual, was directly related to the organisation level and varied over a wide range, from 1.2 × 10^−6^ cm^6^ g^−1^ h^−1^ at cell level to 3 × 10^+3^ cm^6^ g^−1^ h^−1^ at plot level. *κ* was proportional to individual mass, consistent with the findings of many studies on fruit reporting a link between fruit size and pedicel sectional area assumed to be related to transport capacity (Bustan et al., 1995; Ogawa et al., 2007). More specifically, *κ* was proportional to *M*^0.74^, which is quite close to the *M*^0.75^ predicted by the theory of allometric scaling for xylem conductance (Enquist, 2002). Parameter *κ* can be interpreted as a scaling factor dependent on the individual mass associated with a given level of organisation. However, it was not strongly correlated with the degree of density dependence observed in our data.

Parameter α determines the dependence of conductance on population size. Its values were close to zero at the seed level. The hormones produced by seeds are known to play a major role in the formation of the fruit vasculature (Nitsch, 1970; Drazeta et al., 2004), but the very low value obtained for parameter *α* indicates that phloem conductance increases only slowly with seed numbers, potentially explaining the strong density dependence observed at seed level. We found a negative correlation between density dependence and *α* only when the plant level was not included in the statistical analysis. The plant level is a special case because sugar concentration *C* in the plant decreases with increasing plant density, potentially accounting for the strong density dependence at high values of *α*.

We assumed that the maximal unloading rate *V_m_* could vary with species and organisation level. It is at least partly controlled by sugar carriers, which are known to be regulated by endogenous sugar levels (Beckles et al., 2012) and are sensitive to stressors (Ma et al., 2019). The maximal unloading rate varies with genotype within species (Constantinescu et al., 2016). Our results clearly show that density dependence decreases with decreasing the maximal unloading rate per unit dry mass of an individual (*V_m_*). Among others, the cell and fruit levels had a lower density dependence than the seed and plant levels due to a lower *V_m_*. Indeed, when *V_m_*, which is related to potential sink strength, is low, the concentration of sugar in the fruit phloem may greatly exceed *K_m_* (especially if *K_m_* is low) and, according to Equation 2, the unloading rate per individual is then maximal regardless of population size. Consistent with these considerations, our results for peach cells show that a high concentration of sugar in the fruit phloem (*C_f_*) is obtained at low fruit load, leading to density independence with a maximum and constant cell size. A similar result was obtained with the DDM by increasing sugar concentration in the plant phloem (*C*), consistent with the interpretation presented by Lázaro and Larrinaga (2018) of their results on the trade-off between size and seed number.

### Concluding Remarks

We successfully used a modelling approach to demonstrate and explain strong density dependence of fruit unit masses at the seed and plant levels, consistent with previous results. We also showed that these effects were related to phloem conductance and to the maximal unloading rate *V_m_*, and that these relationships were dependent on the sugar concentration in the plant phloem.

Additional studies are required to improve our understanding of the sources of variation in carrier activity and, thus, density-dependence relationships in fruits. Negative relationships between size and number of individuals are frequently reported for other fruit species, including dry fruits (Greenway and Harder, 2007; Labra et al., 2017). The approach developed here could, therefore, be extended to a large range of species.

## Data Availability Statement

The original contributions presented in the study are included in the article/Supplementary Material, further inquiries can be directed to the corresponding author.

## Author Contributions

MG and FL designed the research, implemented the model, performed model simulations and subsequent analyses, and wrote the first version of the manuscript. NB collected some of the data. NB and GV revised and edited the manuscript. All authors contributed to the article and approved the submitted version.

## Conflict of Interest

The authors declare that the research was conducted in the absence of any commercial or financial relationships that could be construed as a potential conflict of interest.

## Publisher’s Note

All claims expressed in this article are solely those of the authors and do not necessarily represent those of their affiliated organizations, or those of the publisher, the editors and the reviewers. Any product that may be evaluated in this article, or claim that may be made by its manufacturer, is not guaranteed or endorsed by the publisher.

## Footnotes

^1^ http://www.tela-botanica.org/page:eflore_bdtfx?referentiel=bdtfx&niveau=2&module=fiche&action=fiche&num_nom=53578&type_nom=nom_scientifique&nom=prunus%20persica
