# Supplementary material for: Resource Translocation Modelling Highlights Density-Dependence Effects in Fruit Production at Various Levels of Organisation
Source: Front Plant Sci. 2022 Jul 8;13:931297. doi: 10.3389/fpls.2022.931297 (PMC9305715; doi:10.3389/fpls.2022.931297)
Supplement: Supplementary file 1 [file Data_Sheet_1.docx]

Supplementary Material

# Supplementary Figures and Tables

## Supplementary Figures


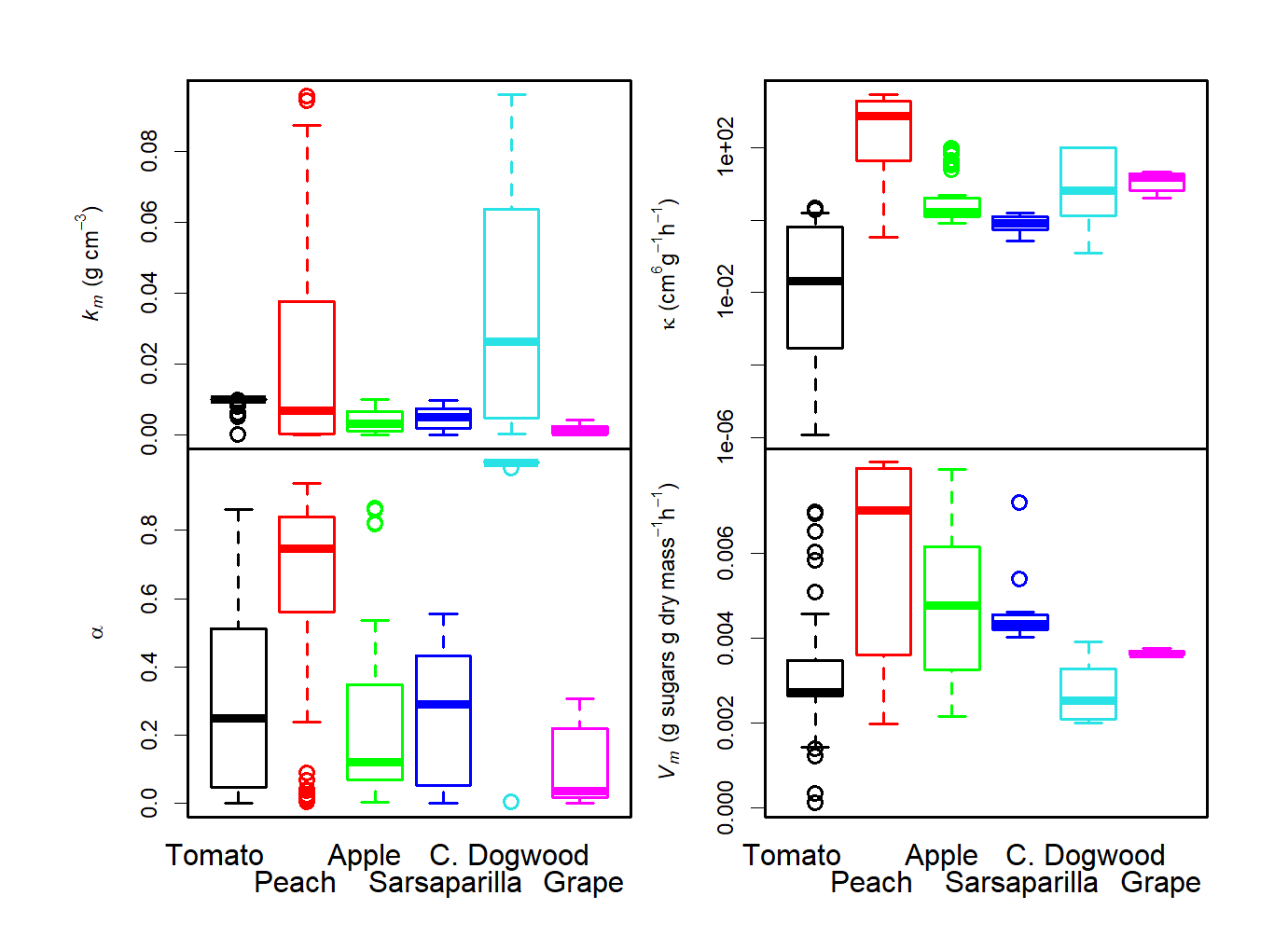


**Supplementary Figure 1.** Box-plot of the estimated values of DDM parameters for each species. A logarithmic scale was used for parameter κ.

**Supplementary Figure 2.** Variation of sugar concentration at plant level (C) as a function of plant density, according to the equation $C=\frac{0.1}{1+e^{b\left( n-d \right)}}$ , where n is plant density, b=1.587 x 10^-3^ and d=2.335 x 10^+3^.

**Supplementary Figure 3.** Temporal change in the concentration of assimilates in the sink phloem (*C_f_*) of peach calculated by the DDM, as a function of the number of cells. The red bold line indicates the *K_m_* value and the areas of low and high fruit load are separated by red dashed lines.

**Supplementary Figure 4.** DDM-modelled cell mass of peach and tomato mesocarps at fruit maturity against the number of cells in the fruit mesocarp for different sugar concentrations (*C*) in plant phloem.

**Supplementary Figure 5.** Density dependence index (DD) for five levels of organisation. The data used are independent from those of this study and are described by Lescourret and Génard (2003), Quilot and Génard (2008) and Prudent et al. (2014).


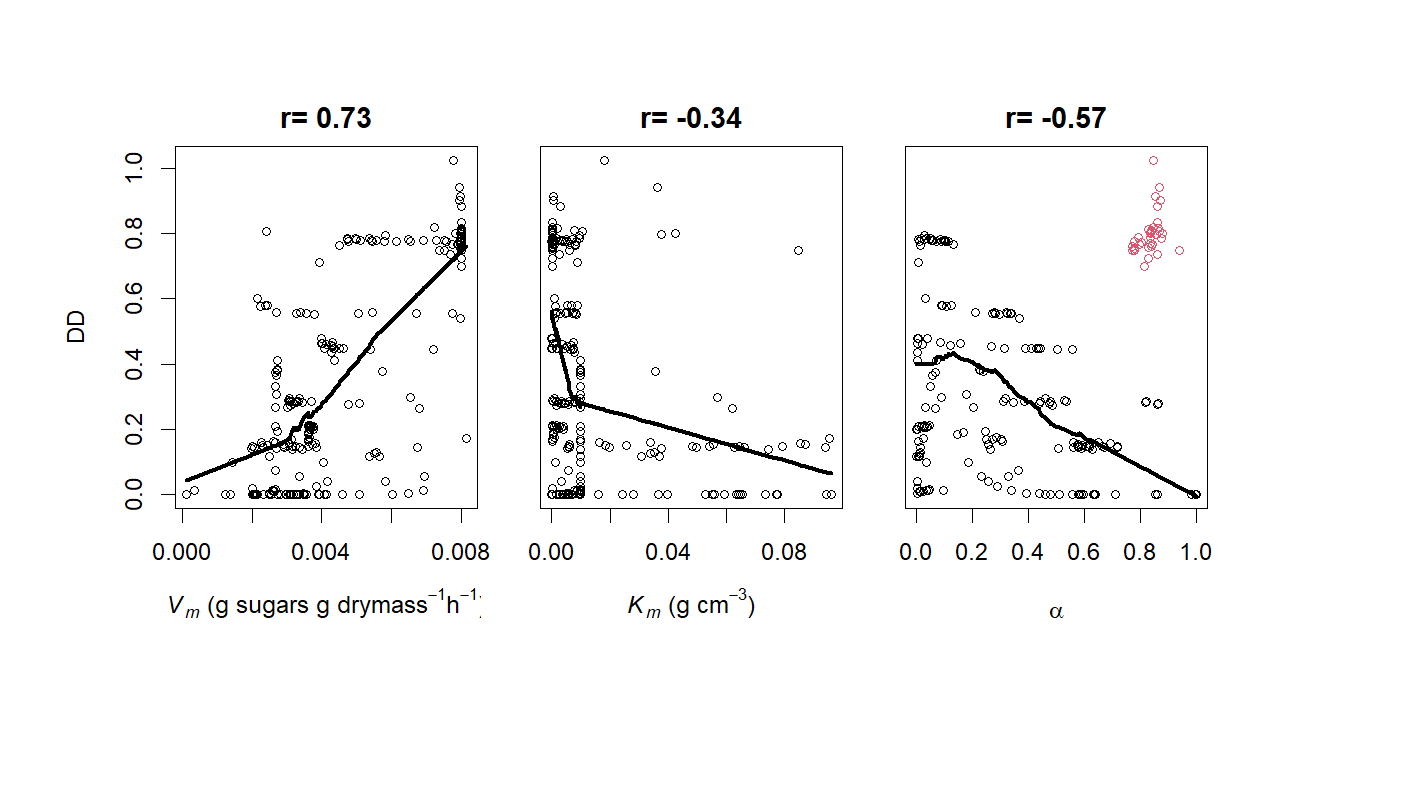


**Supplementary Figure 6.** Variation of DDM-modelled density dependence index (DD) with sugar unloading parameters and α. The Pearson correlation coefficient (r) is shown above the graph for each parameter. The bold lines are smoothing lines. In the case of α, the plant level (red circles) is not considered in the calculation of r or the smoothing line.

**Supplementary Figure 7.** Measured (circles) and DDM-simulated (lines) individual fruit masses at maturity (a) and simulated phloem concentration in the fruit for different numbers of days after anthesis (b) against the number of fruits in clusters of common dogwood berries. The red bold line indicates the *K_m_* value for sugar unloading (eqn 2). Plots c and d were generated from the same simulations as were used for plots a and b, except that the value of *V_m_* was increased by 30%.

## Supplementary Tables

**Supplementary Table 1**. Effect of level of organisation and species on DDM parameter values and density-dependence index (DD) in variance analysis.

Df Sum Sq Mean Sq F value Pr(>F)

_______________________________________________________________

*V_m_*

Level of organisation 4 0.0006158 1.540e-04 111.15 < 2e-16

Species 4 0.0000748 1.871e-05 13.51 6.38e-10

Residuals 234 0.0003241 1.390e-06

*k_m_*

Level of organisation 4 0.01846 0.004616 14.75 9.22e-11

Species 4 0.03257 0.008142 26.02 < 2e-16

Residuals 234 0.07322 0.000313

*κ*

Level of organisation 4 2919.6 729.9 157.28 <2e-16

Species 4 562.2 140.6 30.29 <2e-16

Residuals 234 1085.9 4.6

α

Level of organisation 4 16.460 4.115 159.72 <2e-16

Species 4 9.154 2.289 88.83 <2e-16

Residuals 234 6.029 0.026

*DD*

Level of organisation 4 18.675 4.669 656.7 <2e-16

Species 4 1.331 0.333 46.8 <2e-16

Residuals 234 1.664 0.007

_______________________________________________________________

**Supplementary Table 2**. Parameter κ is related to individual mass at fruit maturity (M) by the relationship κ=1M^0.74^, at all levels of organisation.

________________________________________________________________

Non-linear estimation with the nls function of R software

Formula: k ~ M^a

Parameters:

Estimate Std. Error t value Pr(>|t|)

a 0.739497 0.001442 512.8 <2e-16 ***

Residual standard error: 184.2 on 242 degrees of freedom

Number of iterations to convergence: 6

Achieved convergence tolerance: 2.558e-06

________________________________________________________________

**Supplementary Table 3**. Values of DDM parameters corresponding to the smallest RRMSE for each dataset.

Organisation Species *V_m_  K_m_* κ α

Level (g sugars (g cm^-3^) (cm^6^ g^-1^ h^-1^)

g dry mass^-1^

h^-1^)

________________________________________________________________

Cell Tomato 0.0035 0.01 4e-05 0.63868

Cell Tomato 0.0041 1e-05 2.7e-05 0.63053

Cell Tomato 0.0033 1e-05 2.8e-05 0.58311

Cell Peach 0.004 0.00119 11.05 0.03561

Seed Apple 0.0054 0.00315 1.548 0.05664

Seed Apple 0.0024 0.00872 3.480 0.09136

Seed Sarsaparilla 0.0043 0.00021 0.536 0.42886

Fruit-in-cluster Common Dogwood 0.002 0.00022 0.124 0.00159

Fruit-in-cluster Grape 0.0037 0.00142 16.30 0.02741

Fruit-in-plant Apple 0.0031 0.00037 24.44 0.52961

Fruit-in-plant Peach 0.002 0.00526 84.94 0.50726

Plant Peach 0.0074 0.08479 1748.55 0.93818

________________________________________________________________

**Supplementary Table 4.** Effect of fruit age and position within the truss on cell and fruit masses of tomato (Raissa cv), assessed by analysis of variance, for measured data and DDM predictions.

___________________________________________________________

Measured cell mass

Df Sum Sq Mean Sq F value Pr(>F)

Days after anthesis (DAA) 1 890.6 890.6 206.785 <2e-16

Fruit position 2 0.1 0.0 0.011 0.989

DAA:Fruit position 2 1.8 0.9 0.214 0.808

Residuals 51 219.7 4.3

-----------------------------------------------------------

Predicted cell mass

Df Sum Sq Mean Sq F value Pr(>F)

Days after anthesis (DAA) 1 780.0 780.0 6698.858 < 2e-16

Fruit position 2 1.3 0.7 5.768 0.00552

DAA: Fruit position 2 0.2 0.1 0.900 0.41288

Residuals 51 5.9 0.1

-----------------------------------------------------------

Measured fruit mass

Df Sum Sq Mean Sq F value Pr(>F)

Days after anthesis (DAA) 1 69187 69187 262.259 < 2e-16

Fruit position 2 2854 1427 5.409 0.00741

DAA: Fruit position 2 196 98 0.372 0.69114

Residuals 51 13454 264

-----------------------------------------------------------

Predicted fruit mass

Df Sum Sq Mean Sq F value Pr(>F)

Days after anthesis (DAA) 1 62782 62782 776.954 < 2e-16

Fruit position 2 1973 986 12.208 4.65e-05

DAA: Fruit position 2 102 51 0.634 0.535

Residuals 51 4121 81

___________________________________________________________

**Supplementary Table 5.** Effect of fruit age and fruit load on cell, fruit and truss masses of tomato (Levovil cv), assessed by analysis of variance, for measured data and DDM predictions.

_____________________________________________________________

Measured cell mass

Df Sum Sq Mean Sq F value Pr(>F)

Days after anthesis (DAA) 1 210.52 210.52 205.470 < 2e-16

Fruit load 1 15.61 15.61 15.240 0.000346

DAA: Fruit load 1 1.07 1.07 1.041 0.313653

Residuals 41 42.01 1.02

-------------------------------------------------------------

Predicted cell mass

Df Sum Sq Mean Sq F value Pr(>F)

Days after anthesis (DAA) 1 205.80 205.80 2478.34 < 2e-16

Fruit load 1 14.46 14.46 174.10 2.40e-16

DAA: Fruit load 1 4.11 4.11 49.46 1.48e-08

Residuals 41 3.40 0.08

-------------------------------------------------------------

Measured fruit mass

Df Sum Sq Mean Sq F value Pr(>F)

Days after anthesis (DAA) 1 26383 26383 264.18 < 2e-16

Fruit load 1 5247 5247 52.54 7.36e-09

DAA: Fruit load 1 1964 1964 19.66 6.77e-05

Residuals 41 4095 100

-------------------------------------------------------------

Predicted fruit mass

Df Sum Sq Mean Sq F value Pr(>F)

Days after anthesis (DAA) 1 26864 26864 472.12 < 2e-16

Fruit load 1 5254 5254 92.33 4.67e-12

DAA: Fruit load 1 2853 2853 50.14 1.27e-08

Residuals 41 2333 57

-------------------------------------------------------------

Measured truss mass

Df Sum Sq Mean Sq F value Pr(>F)

Days after anthesis (DAA) 1 328759 328759 153.80 1.85e-15

Fruit load 1 172524 172524 80.71 3.09e-11

DAA: Fruit load 1 22750 22750 10.64 0.00223

Residuals 41 87639 2138

-------------------------------------------------------------

Predicted truss mass

Df Sum Sq Mean Sq F value Pr(>F)

Days after anthesis (DAA) 1 315052 315052 368.08 < 2e-16

Fruit load 1 172536 172536 201.58 < 2e-16

DAA: Fruit load 1 14052 14052 16.42 0.000221

Residuals 41 35093 856

_____________________________________________________________

**Supplementary Table 6.** Effect of fruit age and fruit load on cell, fruit and truss masses of tomato (Cervil cv), assessed by analysis of variance, for measured data and DDM predictions.

_____________________________________________________________

Measured cell mass

Df Sum Sq Mean Sq F value Pr(>F)

Days after anthesis (DAA) 1 8.297 8.297 177.332 2.3e-14

Fruit load 1 0.696 0.696 14.872 0.000544

DAA: Fruit load 1 0.014 0.014 0.304 0.585242

Residuals 31 1.450 0.047

-------------------------------------------------------------

Predicted cell mass

Df Sum Sq Mean Sq F value Pr(>F)

Days after anthesis (DAA) 1 13.944 13.944 1007.09 < 2e-16

Fruit load 1 0.725 0.725 52.40 3.84e-08

DAA: Fruit load 1 0.533 0.533 38.49 6.87e-07

Residuals 31 0.429 0.014

-------------------------------------------------------------

Measured fruit mass

Df Sum Sq Mean Sq F value Pr(>F)

Days after anthesis (DAA) 1 16.526 16.526 119.19 3.78e-12

Fruit load 1 2.534 2.534 18.27 0.000169

DAA: Fruit load 1 0.035 0.035 0.25 0.620386

Residuals 31 4.298 0.139

-------------------------------------------------------------

Predicted fruit mass

Df Sum Sq Mean Sq F value Pr(>F)

Days after anthesis (DAA) 1 28.823 28.823 1974.31 < 2e-16

Fruit load 1 2.427 2.427 166.25 5.38e-14

DAA: Fruit load 1 1.268 1.268 86.82 1.68e-10

Residuals 31 0.453 0.015

-------------------------------------------------------------

Measured truss mass

Df Sum Sq Mean Sq F value Pr(>F)

Days after anthesis (DAA) 1 1718 1718 76.47 7.13e-10

Fruit load 1 6927 6927 308.31 < 2e-16

DAA: Fruit load 1 986 986 43.90 2.10e-07

Residuals 31 696 22

-------------------------------------------------------------

Predicted truss mass

Df Sum Sq Mean Sq F value Pr(>F)

Days after anthesis (DAA) 1 2382 2382 916.2 < 2e-16

Fruit load 1 5579 5579 2146.0 < 2e-16

DAA: Fruit load 1 613 613 236.0 4.83e-16

Residuals 31 81 3

______________________________________________________________

# References

Lescourret, F., and Génard, M. (2003). A multi-level theory of competition for resources applied to fruit production. *Ecoscience* 10, 334–341.

Prudent, M., Dai, Z. W., Genard, M., Bertin, N., Causse, M., and Vivin, P. (2014). Resource competition modulates the seed number-fruit size relationship in a genotype-dependent manner: A modeling approach in grape and tomato. *Ecological Modelling* 290, 54–64. doi: 10.1016/j.ecolmodel.2013.10.023.

Quilot, B., and Genard, M. (2008). Is competition between mesocarp cells of peach fruits affected by the percentage of wild species genome? *Journal of Plant Research* 121, 55–63. doi: 10.1007/s10265-007-0125-9.

## 
